# Hyperbaric oxygen therapy fails to reduce hydrocephalus formation following subarachnoid hemorrhage in rats

**DOI:** 10.1186/2045-9912-4-12

**Published:** 2014-07-09

**Authors:** Qin Hu, Alexander Vakhmjanin, Bo Li, Jiping Tang, John H Zhang

**Affiliations:** 1 Department of Physiology and Pharmacology, Loma Linda University School of Medicine, 11041 Campus Street, Risley Hall, Room 219, Loma Linda CA 92354, USA; 2 Department of Neurosurgery, Loma Linda University School of Medicine, 11041 Campus Street, Risley Hall, Room 219, Loma Linda CA 92354, USA

**Keywords:** Hyperbaric oxygen, Hydrocephalus, Neurobehavioral, Subarachnoid hemorrhage

## Abstract

**Background & purpose:**

Approximately 40% of hemorrhagic stroke survivors develop hydrocephalus. Hyperbaric oxygen (HBO) has been shown to be anti-inflammation following experimental stroke; however, its effect upon post-hemorrhagic hydrocephalus formation is not known. The objective of this study is to investigate whether HBO therapy can effectively reduce hydrocephalus formation and improve neurobehavioral functions in a rat model of subarachnoid hemorrhage (SAH).

**Method:**

Thirty-eight male Sprague–Dawley rats (300-320 g) rats survived for 21 days from SAH by endovascular perforation or sham surgery were used. At 24 hours after SAH, HBO (3 atmospheres absolute) or normobaric oxygen (NBO) administrated for 1 hour once daily for a total of 7 days. Wire hanging and rotarod testing were conducted at 14 days after SAH, and cognitive functions were evaluated via the Morris water maze, between day 17 to day 21 after surgery. At day 21, rats were sacrificed and cerebroventricular volumes were measured histologically.

**Results:**

Hydrocephalus exacerbated neurological deficits after SAH, and HBO multiple treatment tendentially improved the neurobehavioral functions. Spatial learning and memory deficits were noticed after SAH, and rats with hydrocephalus showed worse learning and memory abilities and HBO treatment showed a minor improvement. In the SAH group (room air) 4 rats showed an increased ventricular volume at day 21 after SAH-induction (n = 10). HBO or NBO therapy did not alter the occurrence of hydrocephalus after SAH, as 4 rats in each of these groups showed an increased ventricular volume (n = 10 per group).

**Conclusion:**

Multiple HBO therapy does not ameliorate hydrocephalus formation in a rat model of SAH; however, HBO tendentially improved the neurological functions and spatial learning and memory abilities in rats with hydrocephalus.

## Introduction

Hydrocephalus defines an expansion of the cerebroventricular system, which is associated with a decrease in brain tissue as well as compromised neurological functions. The neurological deficits resulted from hydrocephalus range from subtle neurocognitive and gait problems to severe disability requiring permanent cerebrospinal fluid (CSF) diversion. Approximately 40% of hemorrhagic stroke survivors develop a hydrocephalus [1]. Hydrocephalus can be classified into communicating, noncommunicating or obstructive types [2]. Decreased absorption of CSF at the arachnoid granulations is defined as communicating hydrocephalus and an anatomic obstruction, as noncommunicating. Subarachnoid hemorrhage (SAH) is a common cause of chronic communicating hydrocephalus in clinic, which affects up to 20% of the survivors [3].

Although the cause of chronic hydrocephalus after SAH is still uncertain, previous studies suggested that proliferation of arachnoid cells and leptomeningeal fibrosis, triggered by an inflammatory reaction or blood clotting products, may impair CSF flow through the arachnoid villi, resulting in hydrocephalus [4,5]. Current therapies for hydrocephalus rely on invasive procedures that are associated with high failure and complication rates [6]. And so far no medical treatment is effective in long-term treatment of chronic hydrocephalus. HBO has been shown to reduce inflammation in many chronic diseases [7,8] and proved to be neuroprotective and anti-inflammation following experimental stroke [9]; however, its effect upon post-hemorrhagic hydrocephalus formation is not known. The objective of this current study is to investigate whether multiple HBO therapy can effectively reduce hydrocephalus formation and improve the neurological functions in a rat model of SAH.

## Materials and methods

This study was carried out in strict accordance with the recommendations in the Guide for the Care and Use of Laboratory Animals of the National Institutes of Health. All protocols were approved by the Institutional Animal Care and Use Committee of Loma Linda University.

### Experiment design

This is a retrospective study. A total of 38 adult male Sprague–Dawley rats (300-320 g, Harlan, Indianapolis, IN) survived for 21 days from SAH by endovascular perforation (n = 45) or sham surgery (n = 8) were used for the study. 24 hours after SAH, rats were randomly divided into 3 groups: SAH, SAH with HBO treatment (SAH + HBO), SAH with normobaric oxygen (NBO) treatment (SAH + NBO). Foot-fault, wire hanging and rotarod tests were conducted at post-operative day 14, and cognitive functions were evaluated via the Morris water maze, between day 17 to day 21 after surgery. At day 21, rats were sacrificed after measuring the body weight and cerebroventricular volumes were measured histologically. In SAH, SAH + HBO and SAH + NBO groups, animals were divided into SAH without hydrocephalus (SAH w/t HyC) and SAH with hydrocephalus (SAH/HyC) respectively according to the results of cerebroventricular volume. Data were analyzed in Sham, SAH w/t HyC, SAH/HyC, SAH/HyC + HBO, SAH/HyC + NBO groups.

### Experimental model of SAH and HBO treatment

The endovascular perforation model of SAH was performed as previously described [10]. Briefly, rats were anesthetized with 3% isoflurane in 70%/30% medical-air/oxygen, intubated and kept on artificial ventilation during surgery. Body temperature was monitored by a rectal probe and normothermia was maintained by a heating lamp. A sharpened 4–0 nylon suture was introduced into the right internal carotid artery (ICA) until resistance was felt (approximately 18 mm from the common carotid bifurcation). The suture was then advanced until the resistance was overcome to perforate the bifurcation of the anterior and middle cerebral arteries. The suture was withdrawn immediately after perforation. In Sham operated animals the suture was inserted into the right ICA, however no vessel perforation was performed. After suture removal the incision was closed, and rats were individually housed in heated cages until recovery.

24 hours after SAH, rats were pressurized in a research hyperbaric chamber (1300B; Sechrist) at 3 atmospheres absolutes (ATA) with 100% oxygen (flow of 22 L/min) for 1 h once daily for consecutive 7 days. Doses of HBO were selected based on previous studies [11]. SAH rats treated with NBO were used as a control group.

### Neurological scores

At 24 h after SAH, Garcia test was performed by a blinded investigator as previously described with modifications [12]. The scores given to each rat at the completion of the evaluation was the summation of all 6 individual test scores (spontaneous activity, symmetry in the movement of four limbs, forepaw outstretching, climbing, body proprioception, and response to vibrissae touch). The minimum neurological score (most severe deficit) was 3, and the maximum was 18.

### Foot-fault test, wire hanging and rotarod

At day 14 (6 days after the last HBO treatment), foot-fault test, wire hanging and rotarod were measured by an investigator who was blinded to the experimental groups as previously described [13]. In the foot fault test, the rats were placed on a horizontal grid floor for 2 minutes. The foot fault was defined as when the rat inaccurately placed a fore- or hindlimb and it fell through one of the openings in the grid. The number of foot-faults was recorded and was used for the statistical analysis.

The wire hanging test procedure was basically the same as that described by Shabani et al. [14] and is used to assess the muscle strength and balance. Each rat was suspended with both forepaws on a horizontal steel wire 80 cm long, diameter 7 mm. The animal was held in a vertical position when its front paws were placed in contact with the wire. When the rat grasped the wire, it was released, and the latency to fall was recorded with a stopwatch. Rats were randomly tested and each animal was given three trials with a 30 min inter-trial rest interval.

Rotarod was performed at day 14 after SAH to test coordination and balance as previously described [15]. The rotarod consists of a rotating horizontal cylinder (7 cm diameter) that is divided into 9.5-cm-wide lanes. When placed into a lane, an animal must continuously walk forward to avoid falling off the cylinder. Latency to fall off was detected and recorded by a photobeam circuit. Two consecutive trials were administered, in which the cylinder started turning at 5 rpm, and was accelerated by 2 rpm every 5 sec. To control for any potential learning effect due to previous rotarod exposure, an additional set of more difficult trials was added in which the cylinder started turning at 10 rpm, and was accelerated by 2 rpm every 5 sec.

### Morris water maze

At day 17 to day 21 after SAH, Morris water maze was performed in a blinded setup as previously described [15]. In brief, it consists of three trials (cued, spatial, and probe) done over four consecutive days. All trials lasted a maximum of 60 sec. The cued trials have a visible platform above the water level where the animals were allowed to remain on the platform for 10 sec after finding it or being guided to it. The spatial trials have the platform submerged in the water. Once released, they were allowed to swim in search of the platform. Here the time taken to find the platform measured spatial learning. The probe trial; platform is removed completely and rats were allowed to swim again in search of the platform measuring spatial memory.

### Nissl staining and brain ventricular volume

Rats were fatally anesthetized with isoflurane (≥5%) followed by cardiovascular perfusion with ice-cold PBS and 10% formaldehyde. Brains were removed and postfixed/cryoprotected in 10% formaldehyde/30% sucrose for 3 days, embedded in mounting medium and frozen in liquid nitrogen. For all neuropathological analyses 10 μm thick coronal sections were cut on a cryostat (Leica Microsystems LM3050S), mounted on poly-L-lysine-coated slides and Nissl stained. Three Nissl-stained coronal sections at bregma 0 (+/−250 μm) were randomly chosen and photographed under light microscopy. Morphometric analysis of these slides was performed by computer-assisted (Photoshop CS4, Adobe) hand delineation of the ventricles and the total brain area. The relative ventricle area was calculated as ventricle area/total brain area, and took the average of the three slices as the value for each animal.

### Statistical analyses

Data were expressed as the mean ± SEM. The analysis of the data was performed using GraphPad Prism software. Statistical differences among groups were analyzed by using one-way analysis of variance followed by the Turkey method. A value of *p* < 0.05 was taken as significant.

## Results

### SAH rats showed neurological deficits at 24 h and no difference between groups before treatment

**Figure 1:**
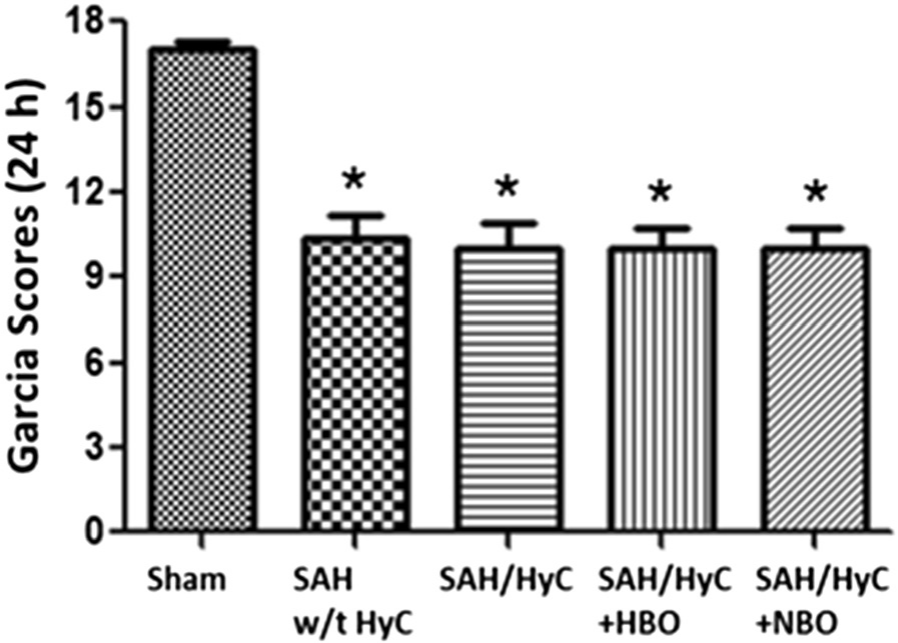
**SAH Rats showed neurological deficits at 24 h and no difference between groups before treatment.** There was a very significant impairment in neurological functions compared with Sham group at 24 hours after SAH. There is no significant difference between SAH w/t HyC, SAH/HyC, SAH/HyC + HBO and SAH/HyC + NBO groups before treatment. Sham, n = 8; SAH w/t HyC, n = 6; SAH/HyC, n = 4; SAH/HyC + HBO, n = 4; SAH/HyC + NBO, n = 4. *p < 0.05 compared with Sham.

At 24 hours after SAH, there was a very significant impairments in neurological functions compared with Sham group (Figure 1, *p* < 0.05, compared with Sham). There is no significant difference between SAH w/t HyC, SAH/HyC, SAH/HyC + HBO or SAH/HyC + NBO groups before treatment. The results showed our SAH model is successful and stable.

### Multiple HBO treatment neither altered the occurrence of hydrocephalus nor affected the ventricular volume after SAH

**Figure 2:**
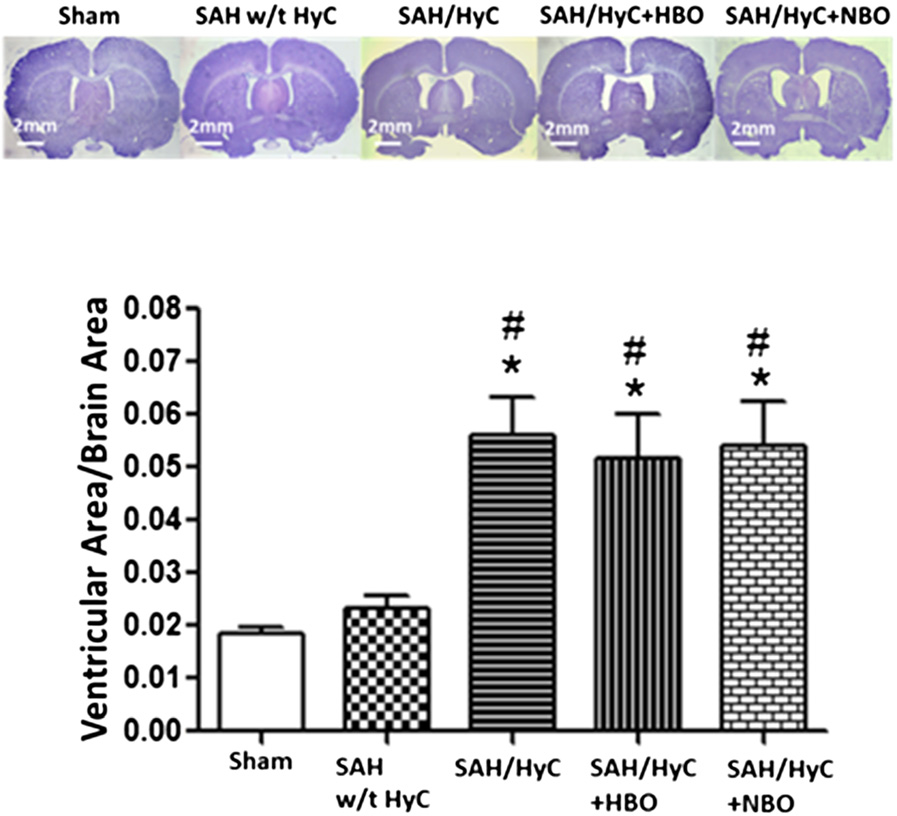
**Multiple HBO treatment neither altered the occurrence of hydrocephalus nor affected the ventricular volume after SAH.** At 21 days after SAH, 4 rats showed an increased ventricular volume compared with Sham; 6 rats showed no significant difference in ventricular volume. HBO or NBO treatment did not alter the occurrence of hydrocephalus, as 4 rats in each of these groups. Neither HBO nor NBO treatment affected the cerebroventricular volumes in the hydrocephalic animals. Sham, n = 8; SAH w/t HyC, n = 6; SAH/HyC, n = 4; SAH/HyC + HBO, n = 4; SAH/HyC + NBO, n = 4. *p < 0.05 compared with Sham, #*p* < 0.05 compared with SAH w/t HyC.

At 21 days after SAH, 4 rats showed an increased ventricular volume compared with Sham (Figure 2, (5.621 ± 0.704)% vs. (1.844 ± 0.129)%, *p* < 0.05 compared with Sham); 6 rats showed no significant difference in ventricular volume (Figure 2, (2.337 ± 0.223)% vs. (1.844 ± 0.129)%, *p* > 0.05 compared with Sham); the occurrence of hydrocephalus is 40%. HBO or NBO treatment did not alter the occurrence of hydrocephalus, as 4 rats (40%) in each of these groups. Multiple HBO treatment did not affect the cerebroventricular volumes in the hydrocephalic animals (Figure 2, (5.181 ± 0.807)% vs. (5.621 ± 0.704)%, *p* > 0.05 compared with SAH/HyC).

### Multiple HBO treatment improved the performance of foot-fault and rotarod in hydrocephalic animals

**Figure 3:**
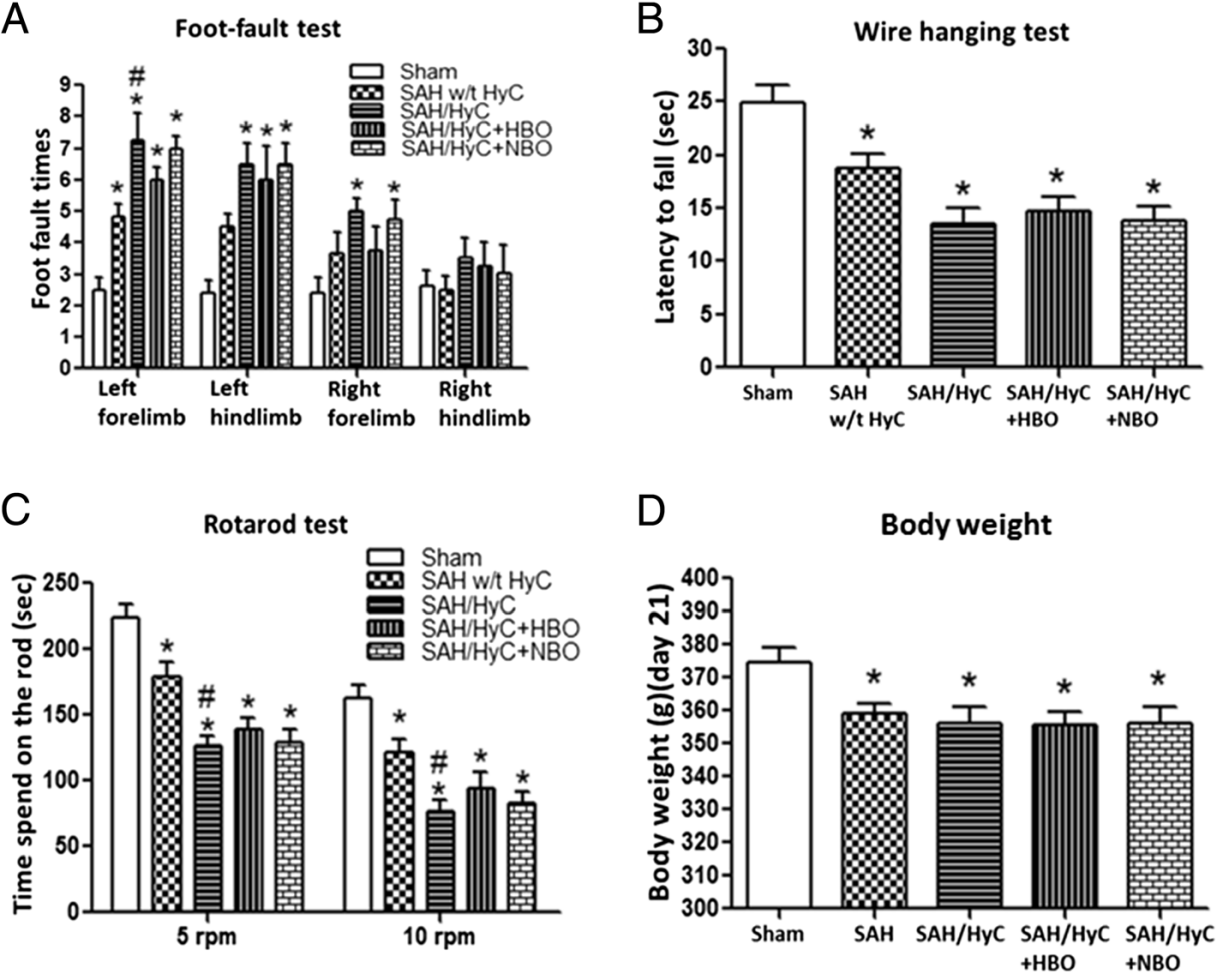
**Multiple HBO treatment mildly improved the neurological deficits in hydrocephalic animals. (A)** 14 days after SAH, the foot fault times in left forelimb were significantly higher than that in Sham group. Hydrocephalus deteriorated the performance, and HBO treatment showed tendency to improve the performance and removed the significance between SAH w/t HyC. **(B)** After SAH, there is no significant reduce of muscle strength between hydrocephalic and non-hydrocephalic animals. HBO treatment did not increase the time of wire hanging. **(C)** In accelerating rotarod, hydrocephalic animals more easily fell off from the rotarod than non-hydrocephalic rats, and HBO treatment showed minor improvement by eliminating the significance between SAH w/t HyC. **(D)** At 21 days after surgery, there is significant difference in body weight between Sham-operated animals and the SAH-operated animals, but no difference between the SAH-operated with HBO or NBO treatment groups. Sham, n = 8; SAH w/t HyC, n = 6; SAH/HyC, n = 4; SAH/HyC + HBO, n = 4; SAH/HyC + NBO, n = 4. *p < 0.05 compared with Sham, #*p* < 0.05 compared with SAH w/t HyC.

At 14 days after SAH, animals showed significant deficits in foot fault in left forelimb than in Sham group (Figure 3A). Hydrocephalus deteriorated the performance (Figure 3A, *p* < 0.05 compared with SAH w/t HyC). HBO treatment did not significantly ameliorate the deficits of foot fault in hydrocephalic animals (Figure 3A, *p* > 0.05, compared with SAH/HyC), but showed tendency to improve the performance and removed the significance between SAH w/t HyC (Figure 3A, *p* > 0.05, compared with SAH w/t HyC). After SAH, there is no significant reduce of muscle strength in hydrocephalic animals (Figure 3B, *p* > 0.05, compared with SAH w/t HyC), and HBO treatment did not increase the time of wire hanging (Figure 3B, *p* > 0.05, compared with SAH w/t HyC). In accelerating rotarod, hydrocephalic animals more easily fell off from the rotarod than non-hydrocephalic rats (Figure 3C, *p* < 0.05, compared with SAH w/t HyC), and HBO treatment showed minor improvement by eliminating the significance between SAH w/t HyC. NBO treatment showed no improvements in the performance foot-fault, wire hanging and rotarod.

There was a significant decrease of body weight at 21 days after SAH (Figure 3D, *p* < 0.05, compared with Sham). Hydrocephalus did not affect the body weight remarkably (Figure 3D, *p* > 0.05, compared with SAH w/t HyC), and HBO treatment has no effect on body weight in long-term (Figure 3D, *p* > 0.05, compared with SAH/HyC).

### Multiple HBO treatment improved spatial learning and memory abilities in hydrocephalic animals

**Figure 4:**
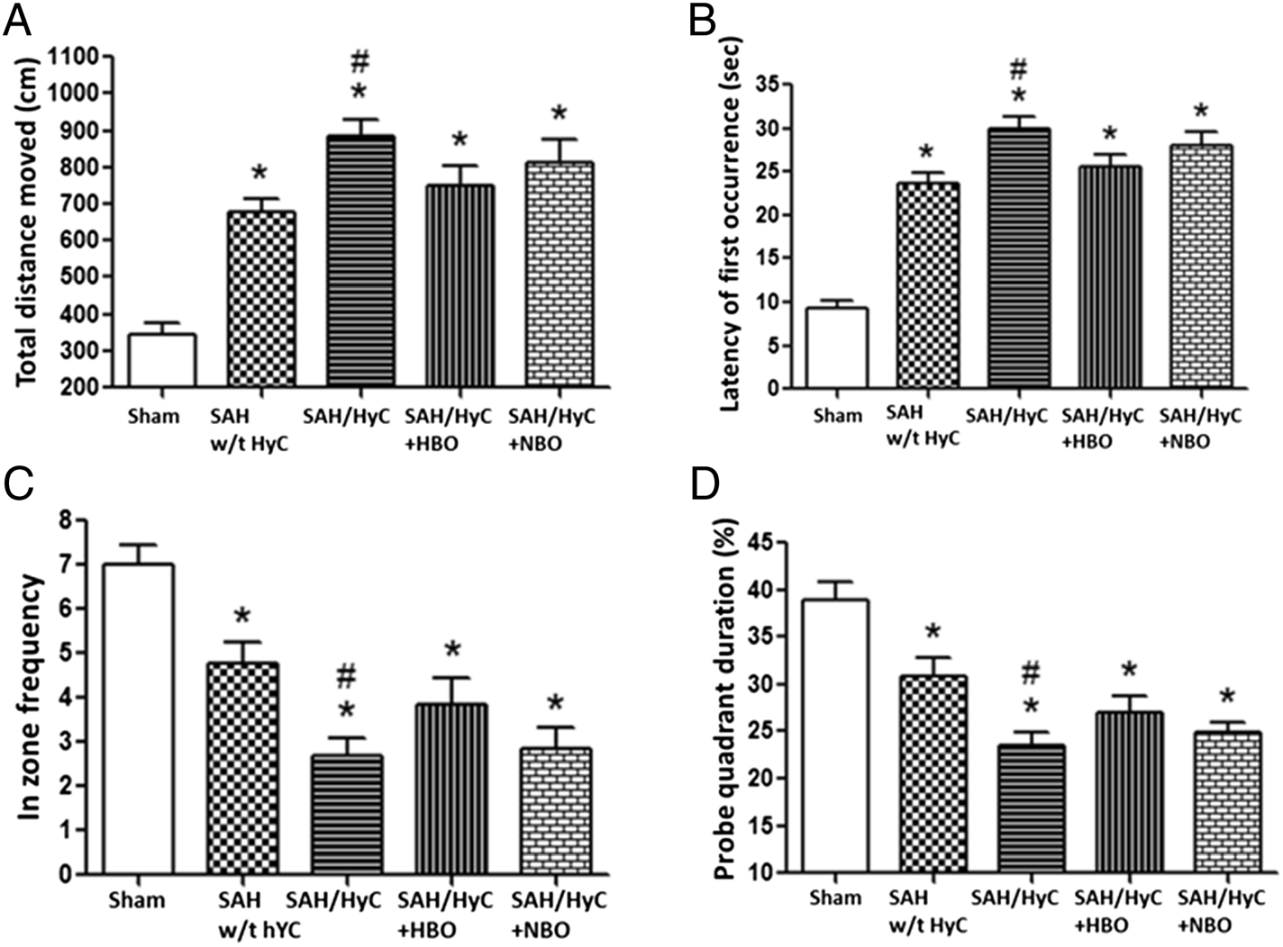
**Multiple HBO treatment improved spatial learning and memory abilities in hydrocephalic animals.** When compared with Sham group, the animals in SAH w/t HyC group had a significantly greater distance moved from the target **(A)**, need more time to reach the platform **(B)**, showed less frequency in target quadrants **(C)**, spent less time in the probe quadrants **(D)**. Hydrocephalus exacerbated the performance. Multiple HBO treatment did not showed significant improvement of memory and learning abilities when compared with SAH/HyC group, but removed the difference between SAH w/t HyC group. Sham, n = 8; SAH w/t HyC, n = 6; SAH/HyC, n = 4; SAH/HyC + HBO, n = 4; SAH/HyC + NBO, n = 4. *p < 0.05 compared with Sham, #*p* < 0.05 compared with SAH w/t HyC.

There were spatial learning and memory deficits at 21 days after SAH. When compared with Sham group, the animals in SAH w/t HyC group had a significantly greater distance moved from the target (Figure 4A), need more time to reach the platform (Figure 4B), showed less frequency in target quadrants (Figure 4C), spent less time in the probe quadrants (Figure 4D). Hydrocephalus exacerbated the performance (Figure 4D, *p* < 0.05, compared with SAH w/t HyC). Multiple HBO treatment did not showed significant improvement of memory and learning abilities when compared with SAH/HyC group (Figure 4, *p* > 0.05 compared to SAH/HyC), but removed the difference between SAH w/t HyC group (Figure 4, *p* > 0.05 compared to SAH w/t HyC).

## Discussion

In the present study, we observed increased ventricular volume and deteriorated neurobehavioral after SAH induced by endovascular perforation in rats. The data showed that 40% of the animals developed hydrocephalus which was associated with ventricular dilation — the typical characteristics of chronic communicating hydrocephalus. The incidence of hydrocephalus is consistent with its clinical relevance and our previous study [16]. Repeated HBO treatment did not ameliorate hydrocephalus formation but tendentially improve the neurological deficits and spatial learning and memory abilities. Our data indicates multiple HBO treatment is beneficial for the recovery of neurological functions after SAH; however, there is no improvement in hydrocephalus.

The treatment of hydrocephalus can be divided into two main groups: surgical and medical treatment [17]. Despite increasing improvements in surgical techniques and instruments, hydrocephalus remains quite a challenge for neurosurgery and there is still a high complication rate. Rapid and effective interventions with medicine are suggested as promising strategies to minimize the occurrence of hydrocephalus. Inflammatory reaction caused by blood clotting products after SAH is claimed to be one of the perpetrators for communicating hydrocephalus [4,18]. HBO has been shown to reduce inflammation in diabetic foot [8], traumatic brain injury [19], and also in experimental stroke [9,20]. HBO treatment can suppress inflammation by reducing neutrophil-endothelial adhesion through S-nitrosation [21], reducing the toll-like receptor signaling pathway [22], increasing heme oxygenase-1 [23] and inhibiting matrix metalloproteinase-9 [24]. In this study, HBO seems invalid to prevent the development of hydrocephalus after SAH. The results suggested that HBO treatment might not work for anti-inflammation when begin at 24 hours after SAH. However, multiple HBO treatment did improve neurobehavioral deficits and spatial learning and memory abilities in long-term in hydrocephalic animals. The improvements of neurological functions and cognitive functions may result from the neuroprotection of HBO and neurogenesis after multiple HBO treatment. HBO treatment has been proved to prevent neuron apoptosis in experimental stroke [25,26]. And our previous studies have demonstrated that delayed and multiple HBO treatment can promote the recovery of neurobehavior through promoting neurogenesis in focal brain ischemic rats [11].

In our study, we observed obviously worse neurological deficits and cognitive functions in hydrocephalic animals than non-hydrocephalic animals at 21 days after SAH. We detected the neurological functions at 24 h after SAH and there is no difference between the SAH operated animals, which excluded the difference in the severity of SAH. These findings support the conclusion that impairments in cognition and motor skills corresponds to ventricular dilation. Previously studies have shown that hydrocephalus is associated with reduction in cerebral blood flow, impairment in myelin production, and progressive loss of periventricular axons [27], and result in progressive motor dysfunction. The impairment in cognition by hydrocephalus is due to the destruction of the fimbria/fornix connections, which is critical for cognitive functions, rather than a direct effect on the hippocampus [28]. However, wire-hanging was not substantially impaired in hydrocephalic animals than non-hydrocephalic animals. And we also have shown hydrocephalus has no effect on body weight gain after SAH. The possible explanation is rats learn to accommodate to their disability quite well [29,30] and wire-hanging might not be a sufficient sensitive indicator of motor and balance. In the rotarad test, we observed a clear difference in the gait and posture, quantifiable gait performance between hydrocephalic animals than non- hydrocephalic animals.

In this study, we didn’t observe beneficial effects of HBO on hydrocephalus in the SAH animals. There are some limitations in this study. First, the sample size was small and it might be not powerful enough to get the statistical significance. Second, we gave HBO treatment at 24 hours after SAH, which is a delayed treatment. It has been reported that HBO is efficient in transient MCAO within the first 6 hours [31] and future studies on the therapeutic window of HBO on hydrocephalus in SAH animals should be investigated. Third, increasing the number of HBO exposures can promote the effects of HBO [32]. In this study we administrated HBO once for each day and multiple-exposure protocols might be more effective.

In conclusion, in the present study we proved that about 40% animals developed to hydrocephalus and showed behavioral and cognitive deficits after SAH. HBO treatment showed minor improvement in neurobehavioral deficits and cognitive functions, and did not affect ventricular volume after SAH. We provide therapeutically relevant data that HBO had no effects on the development of hydrocephalus in SAH model. The improvement in neurological functions may be resulted from the neurogenesis after multiple HBO treatment which has been proved in experimental stroke [11]. This work suggests additional studies should revisit the effective therapy strategies for hydrocephalus in clinical trials after SAH.

## Competing interests

All authors declare that they have no competing interests to disclose.

## Authors’ contributions

QH participated in study design, data analysis and manuscript preparation; AV participated in surgery and data collection; BL participated in data collection and data analysis; JT participated in study design; JHZ participated in study design and manuscript preparation. All authors read and approved the final manuscript.
